# Biomechanical effects of polyaxial pedicle screw fixation on the lumbosacral segments with an anterior interbody cage support

**DOI:** 10.1186/1471-2474-8-28

**Published:** 2007-03-10

**Authors:** Shih-Hao Chen, Ruey Mo Lin, Hsiang-Ho Chen, Kai-Jow Tsai

**Affiliations:** 1 Department of Orthopaedic Surgery, National Cheng Kung University Hospital, Tainan, Taiwan; 2 Department of Biomedical Engineering I-Shou University, Kaohsiung, Taiwan; 3 Department of Orthopaedic Surgery, Cathay General Hospital, Taipei, Taiwan

## Abstract

**Background:**

Lumbosacral fusion is a relatively common procedure that is used in the management of an unstable spine. The anterior interbody cage has been involved to enhance the stability of a pedicle screw construct used at the lumbosacral junction. Biomechanical differences between polyaxial and monoaxial pedicle screws linked with various rod contours were investigated to analyze the respective effects on overall construct stiffness, cage strain, rod strain, and contact ratios at the vertebra-cage junction.

**Methods:**

A synthetic model composed of two ultrahigh molecular weight polyethylene blocks was used with four titanium pedicle screws (two in each block) and two rods fixation to build the spinal construct along with an anterior interbody cage support. For each pair of the construct fixed with polyaxial or monoaxial screws, the linked rods were set at four configurations to simulate 0°, 7°, 14°, and 21° lordosis on the sagittal plane, and a compressive load of 300 N was applied. Strain gauges were attached to the posterior surface of the cage and to the central area of the left connecting rod. Also, the contact area between the block and the cage was measured using prescale Fuji super low pressure film for compression, flexion, lateral bending and torsion tests.

**Results:**

Our main findings in the experiments with an anterior interbody cage support are as follows: 1) large segmental lordosis can decrease the stiffness of monoaxial pedicle screws constructs; 2) polyaxial screws rather than monoaxial screws combined with the cage fixation provide higher compression and flexion stiffness in 21° segmental lordosis; 3) polyaxial screws enhance the contact surface of the cage in 21° segmental lordosis.

**Conclusion:**

Polyaxial screws system used in conjunction with anterior cage support yields higher contact ratio, compression and flexion stiffness of spinal constructs than monoaxial screws system does in the same model when the spinal segment is set at large lordotic angles. Polyaxial pedicle screw fixation performs nearly equal percentages of vertebra-cage contact among all constructs with different sagittal alignments, therefore enhances the stabilization effect of interbody cages in the lumbosacral area.

## Background

Lumbosacral fusion is a relatively common procedure to manage an unstable spine. However, the pseudarthrosis rate may range from 8% to 41% with various surgical techniques, especially when long scoliosis fusions are extended to the sacrum [1-4]. Although pedicle fixation is the most commonly used instrumentation, its role on the lumbosacral junction is questionable. High lordotic angle, large shear force during weight bearing, and short distance of longitudinal rod connector between the L5 and S1 levels have presented the problems including implant failure, loss of reduction, focal kyphosis and pseudarthosis [5].

Glazer [6] et al demonstrated that the rigidity of fixation at the lumbosacral junction may be enhanced by using appropriate anterior interbody fusion techniques. The current interbody fusion cage is expected to share the load transfer, achieve a rigid mechanical support, as well as develop a biological environment to enhance spinal fusion and correct deformity [7-9]. Theoretically, the anterior interbody cage device can restore the disc space height, enlarge the neuroforaminal space, reduce subluxation of the facet joints, preserve load-bearing capacity of the anterior column, and obtain the sagittal plane balance through the spinal segments [9-11]. However, the stand-alone anterior cage significantly reduces intervertebral mobility in flexion and lateral bending, whereas no stabilization is achieved during extension and axial rotation [12-15]. In the clinical setting of fusion at the lumbosacral junction, Christensen et al[16] demonstrated that the circumferential fusion using the wedge-shaped cage and pedicle screws fixation restored lordosis, attained higher union rate, and had a better functional outcome than the instrumented posterolateral fusion.

Pedicle screw systems have been continually modified in design and implantation techniques over the past two decades to reduce the incidence of screw breakage and to improve the accessibility of connecting rod application when they are employed with polyaxial heads [17-20]. The polyaxial head coupling of the pedicle screw introduces a site of reduced compression-bending strength at the screw-rod mount in comparison with monoaxial screw design [19,20]. Theoretically, the combined use of pedicle screws and an interbody cage at the lumbosacral junction has the advantages of construct stabilization, lordosis restoration, and preventing screws breakage [21]. However, there were few reports mentioning the biomechanical performance of the polyaxial versus monoaxial pedicle screw fixation and the associated load transfer mechanism of anterior interbody cage, especially in connection with large lordotic angulation of the pedicle screw-rod linkage.

We designed a synthetic single-level spinal model to investigate the effects of different lordotic angles on the stiffness of the pedicle screw-rod constructs supported with an anterior interbody cage. The first purpose of the present study is to describe the different performances between polyaxial and monoaxial pedicle screws in connection with rod contours of various lordotic angles. The secondary purpose is to analyze the respective effects of polyaxial and monoaxial screws on the overall construct stiffness, cage strain, rod strain, and contact ratio at the vertebra-cage junction.

## Methods

To test the mechanical stability of spinal constructs in relation to various lordotic angles, the biomechanical study was performed on a synthetic model simulating the spinal motion segment, which is composed of two ultrahigh molecular weight polyethylene (UHMWPE) blocks modified from Cunningham et al [22]. In the experiment, there was an anterior wedged cage placed between the UHMWPE blocks. One group used monoaxial screws linked with pre-bent rods of various angles to form the spinal constructs, and the other group adapted polyaxial screws to form the constructs.

**Figure 1:**
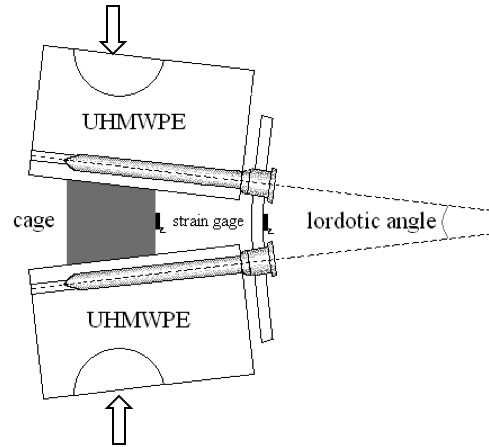
Synthetic spinal models with a varied lordotic angle. The arrows means the direction of compression force.

Experiment: The synthetic model was posteriorly instrumented with four titanium polyaxial or monoaxial pedicle screws (6.0-mm diameter, 40-mm length; Mathys Co, Bettlach, Switzerland), and connected with two rods (5.0-mm diameter) to build the testing constructs. The torque of insertion for each pedicle screw was set at 4.34 Nm, and the design of polyaxial screws was allowed ± 25° of motion for the screw-rod mounting. For each construct, the rod contours were set at four different lordotic configurations (0°, 7°, 14°, and 21°) for the alignment between the screws. A rod of 7°, 14°, or 21° lordotic configurations was pre-bent each based on the curvature, which was formed between the lines of two screw insertion points and the center of a circle (Figure 1). A single SynCage (12° wedged, 18-mm height and 22-mm length; Mathys Co, Bettlach, Switzerland) was positioned centrally (20 mm from the anterior midline and lateral edges of the UHMWPE block). The serrated surfaces above and below the SynCage offering high friction on the interface ensured no sliding of the cage on the block. Once the UHMWPE blocks, as the vertebral endplates, contacted with the upper and lower edges of posterior margin of the SynCage, we tightened nuts for the screw-rod mounting. The torque of coupling the pre-bent rod onto the monoaxial or polyaxial screw head was set at 9.81 Nm. In the monoaxial screws group, the technique of screw-rod mounting was performed along the axis of screw body with a rod adapting to the screw head. In the polyaxial screws group, the technique of screw-rod mounting was performed with a screw holder perpendicular to the rod, while securing the screw head tightly. Consistent biplanar interpedicular distances (40 mm in the coronal plane and 38 mm in the sagittal plane) and a uniform lever-arm distance (40 mm) between the point of anterior load application and the center of the posterior rods were maintained. The segmental lordosis of two blocks was rechecked as 0°, 7°, 14°, or 21° on each construct before compression, flexion, lateral bending and torsion tests. If the lordotic anlgle lost during testing, we applied the construct again.

**Figure 2:**
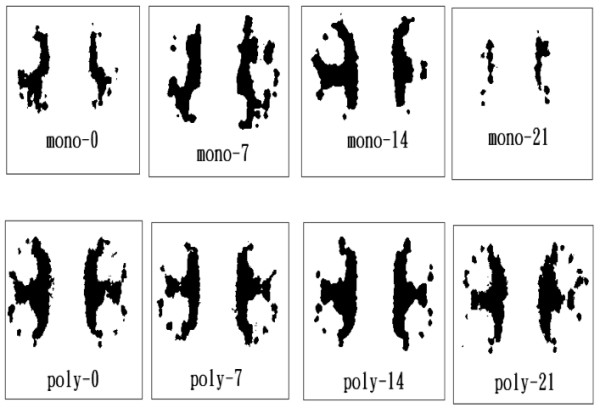
Typical prints for the vertebra-cage contact during compression in all groups (monoaxial or polyaxial screws at 0°, 7°, 14°, or 21° angle of lordotic contour). The original color in the dark area is red to indicate the cage contact.

A uniaxial strain gauge (KFG-1-120-C1-11L1M2R, KYOWA Electronic Instruments Co., Tokyo, Japan) was attached to the posterior part of the cage, and another strain gauge was affixed to the middle of left connecting rod to measure the surface strains of cage and rod separately during the subsequent mechanical testing (Figure 1). During compression and flexion, the surface strain data were obtained from the implants with the sagittally aligned gauges. During lateral bending, the data were obtained from the implants with the coronally aligned gauges [23]. The surface strains on the bent rod and the SynCage were correlated with the applied axial loads. For example, under compression and flexion, we calculated a higher strain data obtained from the posterior surface in the bent rods than in a straight rod, which determined the bending stress of the whole contoured rod during testing. Two prescale Fuji super low pressure films, with a sensitivity range from 0.6 to 2.5 MPa, (Fuji Photo Film Co., Tokyo, Japan) were placed between the contact surfaces of the SynCage and the two UHMWPE blocks to measure the contact area of the cage. The film became red in the compressed area. The red area was scanned with a scanner (HP Scanjet 3570C, HP Co., Palo Alto, CA) to calculate the contact area. The contact ratio was then obtained by dividing the original area of the film with the contact area (Figure 2).

There were four testing steps: compression, flexion, left lateral bending and torsion that were similar to the protocols used by Pavlov et al [21]. For each construct set at each of the four lordotic configurations (0°, 7°, 14°, and 21°), a compressive load of 0–300 N was applied at a displacement rate of 25 mm/min using a testing machine (Q test 10, MTS system Co., Eden Prairie, MN). In the flexion test, a 5 Nm bending moment was loaded 43.5 mm anterior to the center of rotation to make the construct flexed. In the left lateral bending test, a 5 Nm bending moment was loaded to make the construct bend to the left. In the torsion test, 5 Nm of torque was loaded to twist the construct counterclockwise. A cross-link was added transversely across 2 rods to ensure the fixation during torsion. An electric goniometer SG65 (Biometrics Ltd., Gwent, UK) was used to detect the angular motion occurring in the mechanical testing. Every testing step was performed for 8 cycles and the first 3 cycles served as conditioning cycles. A mean of data retrieved from the following 5 cycles and 3 repeated testing experiments were employed for further analysis. Inputs concerning the construct stiffness, surface strain gauges, interface contact ratio performed on the MTS machine was synchronized through a multi-channel signal-conditioning amplifier (InstruNet, GWI, Somerville, MA), and all the data were collected to a personal computer. Compression stiffness of the construct was computed as a ratio of applied load (in Newtons) to linear deformation of the construct (in millimeters). Other rotational stiffness was presented as a ratio of applied torques (in Nm) to linear rotational displacement (in degrees). The surface strain (in microstrain) was recorded at peak load during the fourth loading cycle. Each group involving monoaxial or polyaxial pedicle screws linked with different rod contours has 3 data points for the statistical analysis.

### Statistics

All data were shown as the mean ± standard deviation. Multiple-factor analyses of variance (ANOVA) on the construct stiffness, rod strain, cage strain, and percentage of contact area were conducted to find differences existed among the two instrumentation methods (monoaxial screws with a cage support, and polyaxial screws with a cage support) and the four lordotic rod contours (0°, 7°, 14°, and 21°). The statistical difference between the monoaxial and polyaxial screws groups was calculated by independent t-test. If the p value was less than 0.05, it was defined as significant difference. Statistical analyses were performed with SPSS, Version 10.0 (SPSS, Inc., Chicago, IL).

## Results

### Stiffness

#### Compression stiffness

**Figure 3:**
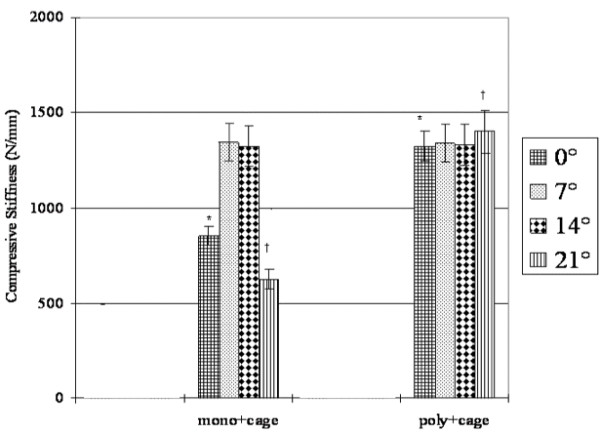
Comparison of compressive stiffness among constructs with various lordotic contours. Two fixation techniques include monoaxial screw with a cage (mono+cage), and polyaxial screw with a cage (poly+cage). † Significant difference (p < 0.05) between mono+cage group and poly+cage group in 21° lordotic angle. * significant difference (p < 0.05) between mono+cage group and poly+cage group in 0° lordotic angle.

In the polyaxial screws group supported with a cage, the compression stiffness of the constructs was not altered by the four different lordotic configurations. However, in the monoaxial screws groups supported with a cage, the compression stiffness was about one half in the constructs linked with 0° and 21° lordotic rod contours compared to those in the constructs linked with 7° and 14° lordotic rod contours (Figure 3). In the constructs linked with 21° lordotic rod contours, the polyaxial screws group had more than twice of the compression stiffness compared to the monoaxial screws group (compression stiffness of the polyaxial screws group = 1402 ± 147 N/mm; compression stiffness of the monoaxial screws group = 626 ± 74 N/mm, p = 0.01).

#### Flexion stiffness

**Figure 4:**
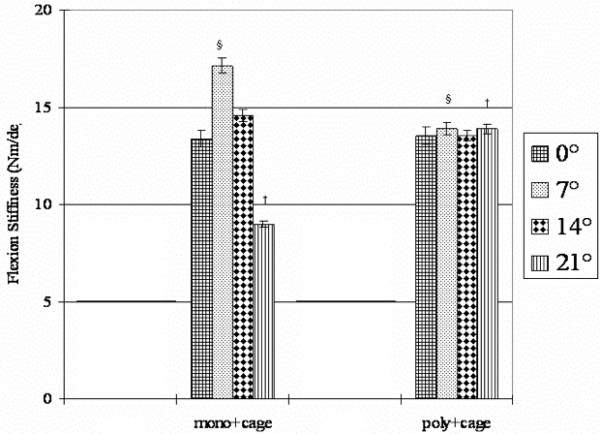
Comparison of flexion stiffness among constructs with various lordotic contours. Two fixation techniques include monoaxial screws with a cage (mono+cage), and polyaxial screws with a cage (poly+cage). † Significant difference (p < 0.05) between mono+cage group and poly+cage group in 21° angle of lordotic contour. § Significant difference (p < 0.05) between mono+cage group and poly+cage group in 7° angle of lordotic contour.

In the polyaxial screws group supported with a cage, the flexion stiffness of the constructs was not altered by the four different lordotic configurations. However, in the monoaxial screws group supported with a cage, the flexion stiffness was about one half in the constructs linked with 21° lordotic rod contours compared to those in the constructs linked with 7° and 14° lordotic rod contours (Figure 4). In the constructs linked with 21° lordotic rod contours, the polyaxial screws group had about 50% more flexion stiffness compared to the monoaxial screws group.

#### Lateral bending stiffness

**Figure 5:**
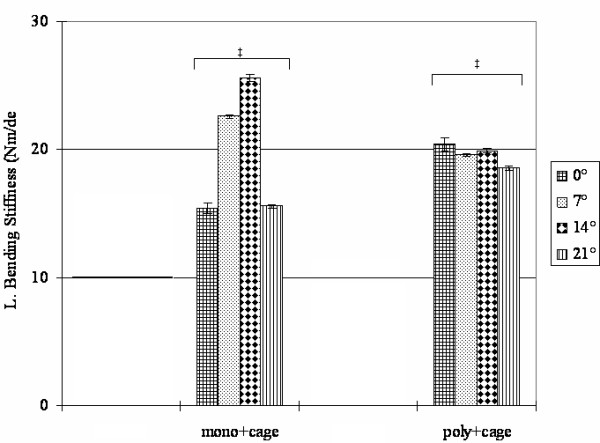
Comparison of lateral bending stiffness among constructs with various lordotic contours. Two fixation techniques include monoaxial screw with a cage (mono+cage), and polyaxial screw with a cage (poly+cage). ‡ Significant difference (p < 0.05) between mono+cage group and poly+cage group in 0°, 7°, 14°, or 21° angle of lordotic contour.

In the polyaxial screws group supported with a cage, the lateral bending stiffness of the constructs was not altered by the four different lordotic configurations. However, in the monoaxial screws group supported with a cage, the lateral bending stiffness was about two-third in the constructs linked with 0° and 21° lordotic rod contours compared to those in the constructs linked with 7° and 14° lordotic rod contours (Figure 5). In the constructs linked with 0° and 21° lordotic rod contours, the polyaxial screws group had about 30% to 40 % more lateral bending stiffness compared to the monoaxial screws group.

#### Torsion stiffness

In torsion, the insertion of a cage did not increase the stiffness of the construct. There is no significant difference between the polyaxial screws group and the monoaxial screws group in torsion stiffness (polyaxial screws with cage: 3.10 ± 0.18 Nm/deg vs. monoaxial screws with cage: 2.91 ± 0.11 Nm/deg). The different lordotic configurations did not show any effect on the torsion stiffness of the two instrumentation methods supported with a cage.

### Contact ratio

**Figure 6:**
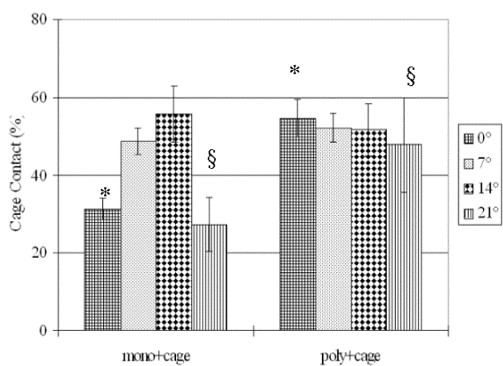
Comparison of cage contact ratio among constructs with various lordotic contours during compression. Effects of monoaxial and polyaxial pedicle screws are illustrated respectively. * significant difference from data of 0° group (p < 0.05). § significant difference from data of 21° group (p < 0.05).

Percentage of vertebra-cage contact was measured as contact ratio between the cage and the UHMWPE blocks under compression. In the monoaxial screws group supported with a cage, the measured contact ratio under compression was significantly lower in the constructs linked with 0° and 21° lordotic rod contours than those in the constructs linked with 7° and 14° lordotic rod contours. However, in the polyaxial screws group supported with a cage, the measured contact ratio under compression did not significantly vary in the constructs of four different lordotic configurations (Figure 6). Averaged cage contact ratio was significantly larger in the polyaxial screw group (51.6 ± 5.3%) than that in monoaxial screws group (40.8 ± 13.7%). Interestingly, in the constructs of 0° and 21° lordotic configurations under compression testing, the polyaxial screws group had 20% more contact ratio than the monoaxial screws group did (Table 1). According to the data above, both 0° and 21° lordotic configuration of the monoaxial screws group had lower contact ratio than others. Therefore, we define the 0° and 21° construct of monoaxial screws as low contact ratio group that may help us to investigate the relationships about the load-sharing of anterior cage and posterior screw/rod complex.

### Rod strain and cage strain

In the testing of compression, flexion and lateral bending, the constructs of low contact ratio (monoaxial screws linked with 0° and 21° rod contours) had significantly higher rod strain than the other constructs (Table 2). On the contrary, these constructs of low contact ratio had lower cage strain than the other constructs (Table 3). However, in the polyaxial screws group supported with a cage, the measured rod and cage strains did not significantly vary in each construct of four different lordotic configurations. Interestingly, in the constructs of 0° and 21° lordotic configurations under compression, flexion, and lateral bending tests, the polyaxial screws group showed more effectively to decrease the rod strain and increase cage strain than monoaxial screws group did. Measurement of the rod strain under torsion was not recorded because the cross-link replaced the strain gauge attachment to the central position of the rod.

## Discussion

In the lumbosacral junction, the anterior interbody cage is recommended in conjunction with pedicle screws fixation for the treatment of high-grade spondylolithesis or long segmental fusion construct to prevent flatback deformity, implant failure and pseudarthosis. In the past, synthetic models had been chosen to analyze the effects of interbody cage positioning within the sagittal plane on the overall construct stiffness [24]. Polly et al [24] described 18 fold increase of construct stiffness by the anterior placement of cage in sagittal plane, cage strain increasing with more-anterior positions, and a single large cage construct being biomechanically compatible with a dual-cage construct. However, the lordotic effect on the spinal segments was not examined as a variant. This study was designed to examine the biomechanical behavior of posterior pedicle-based implants and the associated load transfer mechanism of an anterior interbody cage under the influence of varying lordotic angulations of pedicle screw-rod linkage. The main findings in the current study are as follows: 1) the large segmental lordotic configuration can decrease the stiffness in the monoaxial screws group. 2) polyaxial screws rather than monoaxial screws combined with an interbody cage fixation provide higher compression and flexion stiffness in 21° segmental lordosis; 3) polyaxial screws enhance the contact ratio of the interbody cage in 21° segmental lordosis. Therefore, the high lordotic angle, as in the lumbosacral area, has the negative influence on the stiffness of the posterior pedicle-based screw construct and recommends further supplementary fixation. Polyaxial pedicle screw used among all constructs with an interbody cage support produces nearly equal percentage of cage contact in different sagittal alignments, and affords supplementary stabilization of the interbody cage in an event of large lordotic segments.

The polyaxial head design has made the pedicle screw more adjustable to connect the rod and secure the head to the screw. Fogel et al [18] reported the subtle design variations in the complex locking mechanism of the polyaxial head screw-rod systems, and polyaxial head coupling might contribute to preventing the screw-rod breakage. However, the biomechanical difference between the polyaxial and monoaxial screws is not well understood. The biomechanical stiffness of the pedicle screw constructs has demonstrated the importance of bone-implant interface, bone density, screw diameter, and insertional torque [17,22]. In the current study, a synthetic model was matched with identical bone density, screw diameter, and insertional torque to investigate the various parameters including construct stiffness, rod strain, cage strain, and percentage of cage contact under compression, flexion, lateral bending and axial rotation. We found significantly biomechanical differences among the constructs of low contact ratio (monoaxial screws linked with 0° and 21° rod contours) and the other constructs. In the monoaxial screws group, compression stiffness of constructs with 0° and 21° configurations were significantly lower than those with 7° and 14° configurations. While, in high segmental lordosis (21°), the polyaxial screws group provided better construct stiffness and load-sharing under flexion and compression than the monoaxial screws group did. Theoretically, polyaxial screw systems could stabilize the spinal construct by permitting secure purchase of the screw-rod, accommodating bending stress of the rod on the mobile screw head, transmitting the load to the anterior interbody cage, and equalizing stress distribution on the vertebra-cage interface, even in the event of large lordotic segments at the lumbosacral junction.

The achievement of optimal sagittal alignment to maintain physical functioning of the spine is the major goal of spinal fusion surgery. Recently, the impact of wedge-shaped cage geometry on segmental lordosis and restoration of the disc space height has been demonstrated, also it permits a considerable load transfer through the strong periphery of the endplate and lowers the incidence of subsidence [11,21]. The high contact space can achieve load transfer and bring better compressive stiffness. However, Tsantrizos et al [25] reported no correlation between contact area and compression stiffness in the cadaveric model. Since it is hard to have homogeneous specimen from the cadaveric samples, the current synthetic model may provide a stable environment that can observe single or coupled factors such as segmental lordosis, contact areas and load-sharing of the instrumentation to optimize the construct. Theoretically, in addition to the local bone quality, the vertebra-cage contact ratio, the construct stiffness and the stress on the implants are considered as determining factors of cage subsidence, migration and bone growth [11,12,15,25,26]. The spinal instrumentation is mainly subjected to bending stress, and the load-sharing of the implants decreases concurrently with the development of spinal fusion [23,26]. Takahata et al [26] demonstrated the time-related changes in bending strain of the rod during flexion- extension and lateral bending, and the change reached a plateau whereas maturation of anterior fusion mass. The biomechanical stabilizing trends are also described by Kanayama [23] et al in a sheep posterolateral spinal fusion model. In the current study, the contact area of vertebra-cage, the rod and cage strains are more favored in the polyaxial screws group than the monoaxial screws group especially at a large segmental lordosis (21°). Concurrently, the polyaxial screws group presented higher compression and flexion stiffness than the monoaxial screws group under a wide range of sagittal angulations. Although the integration of the bone graft on load-shielding effect of interbody cages remained undefined, circumferential fusion procedures using polyaxial pedicle screws fixation was favorably accessed in the lumbosacral spine.

Experimental models of in vitro testing of spinal fixation are limited by constraints on availability of cadavers, variations in quality of some structures, such as bone density and area of endplate preparation, implant location and fixation method [22]. In the present study, a synthetic model was chosen to isolate lordosis as one of the primary variables and to replicate the behaviors of pedicle-based implants on a single motion segment with an interbody cage support. When a cage was inserted in the synthetic model with a prepared flat endplate, an axial load was mainly transmitted through the peripheral endplate, resulting in a uniform stress distribution for the parameter measures. Therefore, accepting the model constraints of being short of posterior elements, the authors believe this is one of the best methods available for specifically analyzing the effect of various lordotic angulations on construct stiffness and strain loading of spinal implants. However, we must mention that this testing model does not consider the effects of the facet joint, intervertebral disc and adjacent soft tissues in the normal lumbosacral spine. Also, numerous alternative fixation techniques across the lumbosacral junction, such as iliosacral screws, coupled alar and sacral screws, S1 and S2 pedicle screws, intrasacral rodding, transvertebral L5-S1 fixation, or Luque-Galveston technique, cannot be tested in this synthetic model [6,27].

## Conclusion

Our study using a synthetic model of pedicle-based screw construct with an anterior cage support shows that the polyaxial screws group has a higher contact ratio, compression and flexion stiffness than the monoaxial screws group especially when the large segmental lordosis angle is at 21°. When lordotic rod contour is set at 7° and 14° approaching the angle of wedged cages (12°) in the monoaxial screw groups, the percentage of cage contact for initial stability of the construct is favorable. However, the polyaxial screw system performs nearly equal percentages of the vertebra-cage contact among all constructs with different sagittal alignments, therefore enhances the stabilization effect of interbody cages in the lumbosacral area.

## Competing interests

The author(s) declare that they have no competing interests.

## Authors' contributions

SHC participated in the study desgn, in collecting the data, the statistically analyses and drafting of the manuscript.

RML and HHC participated in the study design.

KJT advised and assisted drafting of the manuscript.

All authors read and approved the final manuscript.

## Pre-publication history

The pre-publication history for this paper can be accessed here:



## Figures and Tables

**Table 1:** Comparison of contact ratio of the vertebra-cage (Mean ± S.D. %) for all constructs

Testing	Contour	Monoaxial screws	Polyaxial screws
Compression	0°	31.3 ± 2.82 †	54.7 ± 4.75†
	7°	48.7 ± 4.21	52.1 ± 2.96
	14°	55.6 ± 8.12	51.6 ± 5.78
	21°	27.3 ± 10.5 ‡	47.8 ± 6.32‡

† Significant difference in monoaxial screws group and polyaxial screws group (p < 0.05).

‡ Significant difference in monoaxial screws group and polyaxial screws group (p < 0.05).

**Table 2:** Comparison of rod strain (Mean ± S.D. μE) for all constructs

Testing	Contour	Monoaxial screws	Polyaxial screws
Compression	0°	893 ± 123 †	172 ± 33†
	7°	248 ± 23 *	154 ± 20*
	14°	268 ± 52 #	168 ± 25#
	21°	873 ± 170 ‡	161 ± 23‡

Flexion	0°	595 ± 34 †	256 ± 20†
	7°	22.1 ± 4.8 *	266 ± 12 *
	14°	46.0 ± 4.4 #	203 ± 27#
	21°	633 ± 45 ‡	162 ± 15 ‡

Lateral Bending	0°	540 ± 33†	404 ± 4†
	7°	258 ± 22*	381 ± 10 *
	14°	273 ± 6 #	430 ± 28 #
	21°	521 ± 16 ‡	407 ± 6‡

† Significant difference between monoaxial screws group and polyaxial screws group (p < 0.05).

* Significant difference between monoaxial screws group and polyaxial screws group (p < 0.05).

# Significant difference between monoaxial screws group and polyaxial screws group (p < 0.05).

‡ Significant difference between monoaxial screws group and polyaxial screws group (p < 0.05).

**Table 3:** Comparison of cage strain (Mean ± S.D. μE) for all constructs

Testing	Contour	Monoaxial screws	Polyaxial screws
Compression	0°	16.9 ± 8.20 †	120 ± 13†
	7°	69.8 ± 19.4 *	129 ± 8*
	14°	141 ± 13	137 ± 14
	21°	32 ± 7.3‡	126 ± 9‡

Flexion	0°	6.30 ± 0.32 †	46.3 ± 4.67†
	7°	32.1 ± 2.44 *	44.1 ± 1.85*
	14°	68.4 ± 4.42 #	45.4 ± 5.65#
	21°	27.3 ± 14.7 ‡	50.8 ± 5.32‡

Lateral Bending	0°	5.71 ± 0.08 †	58.3 ± 3.50†
	7°	32.1 ± 2.00 *	55.4 ± 3.76*
	14°	50.5 ± 6.48	53.0 ± 6.05
	21°	7.18 ± 1.81‡	59.7 ± 2.73‡

† Significant difference between monoaxial screws group and polyaxial screws group (p < 0.05).

* Significant difference between monoaxial screws group and polyaxial screws group (p < 0.05).

# Significant difference between monoaxial screws group and polyaxial screws group (p < 0.05).

‡ Significant difference between monoaxial screws group and polyaxial screws group (p < 0.05).
